# The Delayed-Onset Mechanical Pain Behavior Induced by Infant Peripheral Nerve Injury Is Accompanied by Sympathetic Sprouting in the Dorsal Root Ganglion

**DOI:** 10.1155/2020/9165475

**Published:** 2020-06-16

**Authors:** Pei Liu, Qing Zhang, You-shui Gao, Yi-Gang Huang, Junjie Gao, Chang-qing Zhang

**Affiliations:** ^1^Department of Orthopedic Surgery, Shanghai Jiao Tong University Affiliated Sixth People's Hospital, Shanghai 200233, China; ^2^Shanghai Institute of Materia Medica, Chinese Academy of Sciences, Shanghai 201203, China

## Abstract

**Background:**

Sympathetic sprouting in the dorsal root ganglion (DRG) following nerve injuries had been proved to induce adult neuropathic pain. However, it is unclear whether the abnormal sprouting occurs in infant nerve injury.

**Methods:**

L5 spinal nerve ligation (SNL) or sham surgery was performed on adult rats and 10-day-old pups, and mechanical thresholds and heat hyperalgesia were analyzed on 3, 7, 14, 28, and 56 postoperative days. Tyrosine hydroxylase-labeled sympathetic fibers were observed at each time point, and 2 neurotrophin receptors (p75NTR and TrkA) were identified to explore the mechanisms of sympathetic sprouting.

**Results:**

Adult rats rapidly developed mechanical and heat hyperalgesia from postoperative day 3, with concurrent sympathetic sprouting in DRG. In contrast, the pup rats did not show a significantly lower mechanical threshold until postoperative day 28, at which time the sympathetic sprouting became evident in the DRG. No heat hyperalgesia was presented in pup rats at any time point. There was a late expression of glial p75NTR in DRG of pups from postoperative day 28, which was parallel to the occurrence of sympathetic sprouting. The expression of TrkA did not show such a postoperative syncing change.

**Conclusion:**

The delayed-onset mechanical allodynia in the infant nerve lesion was accompanied with parallel sympathetic sprouting in DRG. The late parallel expression of glial p75NTR injury may play an essential role in this process, which provides novel insight into the treatment of delayed adolescent neuropathic pain.

## 1. Introduction

Neuropathic pain is characterized by spontaneous pain, allodynia, or hyperalgesia and could be rapidly triggered by adult nerve injury. The pain control is still challenging since neuropathic pain after adult nerve injury is mostly poorly relieved by conventional pharmacological treatment [1]. A population-based cohort study observed that neuropathic pain-affected middle-aged persons were more often and peaked between 70 and 79 years of age [2]. In contrast, no evidence of such pain has been reported in infancy, and only very few cases reported before 6 years of age [3–5]. For example, brachial plexus avulsion could induce intense neuropathic pain of up to 70% of adults which is barely seen at birth [4]. The rat models of neuropathic pain demonstrated that persistent mechanical allodynia was not explored in infant rats within 4 weeks after surgery [6]. However, recently, a delayed onset of neuropathic pain syndrome was reported in late childhood and adolescence as a consequence of early-life nerve injury [5, 7]. Consistently, the delayed-onset pain hypersensitivity in the adolescence period was also observed in animals of nerve injury during neonatal period [8–12]. Since infants are able to develop nociception and exhibit inflammatory pain behaviors, the underlying mechanism for this delayed onset of neuropathic pain is remained unknown.

Aberrant sprouting of sympathetic fibers around neuron somas at dorsal root ganglia (DRG) after peripheral nerve injury has been demonstrated to be correlated with adult neuropathic pain behaviors [13–20]. The sympathetic sprouting fibers may increase the expression of *α*-adrenergic receptors, enhance excitability, produce spontaneous activity, and alter the microenvironment of DRG neurons [21–26]. Surgical sympathectomy can alleviate mechanical allodynia induced by spinal nerve lesions [27]. Clinical studies also verified either blockade of sympathetic nerve or intravenous infusion of *α*-adrenergic receptors such as phentolamine could relieve the symptom of sympathetically maintained pain [28, 29]. Although sympathetic sprouting plays such an essential role in the development of nerve injury-triggered pain in adults, it is still unclear whether it is associated with the nerve injury in infants.

In this study, we compared the histological changes in the ipsilateral DRG after L5 spinal nerve ligation (SNL) of pup rats with those of adult rats, focusing on the postoperative time course of sympathetic sprouting fibers. This investigation may provide a new insight into the mechanisms underlying the delayed onset of neuropathic pain in nerve lesions during the neonatal period.

## 2. Materials and Methods

### 2.1. Animal Preparation and Surgery

Animal experiments were performed on male Sprague Dawley rats aged 4 months (weighing 250-300 g) or pups at postnatal day 10 (weighing 22-26 g). All protocols were approved by the Animal Research Committee at Shanghai Institute of Materia Medica, Chinese Academy of Sciences. All animal experiments were carried out following the National Institutes of Health guide for the care and use of laboratory animals (NIH Publications No. 8023, revised 1978). The animals were kept in controlled conditions of temperature (23 ± 2°C), humidity (50 ± 5%), and a 12 h light/dark cycle. The adult rats had free access to sterile food and water, and pups were fed by their mothers.

### 2.2. Surgery

L5 spinal nerve ligation surgery was performed in all adult and pup rats under intraperitoneal anesthesia with 1% pentobarbital sodium (Shanghai Westang Bio-Tech Co., Shanghai, China, 50 mg/kg). The procedure of surgery is previously described by Kim and Chung [30]. After the skin preparation, a longitudinal incision of 5 mm was made lateral (left) to the midline from the level of the L5 vertebra to the first sacral vertebra. The paravertebral muscles were retracted instead of muscle removal, as we previously introduced [31]. The L5 spinal nerve was exposed and ligated to half of its diameter with 6-0 (adult rats) or 7-0 (pups) silk thread and then divided 5 mm distal to the ligation. Much care was taken not to injure the L4 spinal nerve. The sham control animals in both adult and pup rats were performed identically except that the nerves were not ligated after exposure of the L5 spinal nerve. The wound was closed with 4-0 silk thread.

### 2.3. Behavior Test

For both adult rats and pups, there were 6 rats in the sham group and 10 in the SNL group. Behavioral tests for mechanical allodynia and thermal hyperalgesia were performed at 1 day before and 3, 7, 14, 28, and 56 days after surgery. All the behavioral tests were performed by 2 persons who were not aware of the group.

### 2.4. Mechanical Allodynia

The mechanical withdrawal threshold was measured by an electronic von Frey anaesthesiometer (Ugo Basile, Italy). In a quiet room, each rat was placed in a transparent plastic box with a metal grid bottom suspended 50 cm above the laboratory bench top, allowing for 20 minutes of behavioral acclimatization. An electronic von Frey polypropylene tip was applied perpendicularly to the midplantar surface of the hind paw, with a tilted mirror below the grid to provide a clear view of the paw [32]. The threshold pressure was automatically recorded when the paw withdraws, followed by a clear flinch response [33]. Each experiment was repeated 3 times at 5-minute intervals. The mean value was calculated as the mechanical withdrawal threshold.

### 2.5. Heat Hyperalgesia

The thermal hyperalgesia was measured by paw withdrawal latency. The plantar side of the hind paw was placed on the surface of a hot plate (YLS-12A, Shanghai Precision Instruments Co., 50°C) after the adult or pup rats acclimatized to the behavioral apparatus [34, 35]. The latency value for paw withdrawal due to the heat stimulus was manually recorded with a chronometer. Only a clear unilateral withdrawal of the paw was considered. Each experiment for measuring thermal withdrawal latency was repeated three times at 10-minute intervals. The mean value was considered as thermal withdrawal latency.

### 2.6. Immunohistochemical Staining of DRG

Forty rat pups or adults were randomly divided into the sham control (*n* = 15) and L5 SNL group (*n* = 25). On the postoperative day of 3, 7, 14, 28, and 56, 5 rats of SNL group and 3 of the sham control group were perfused with 4% paraformaldehyde for immunohistochemical staining of DRG. Samples were cut in 7 *μ*m thick tissue sections using a cryostat and collected on glass slides for immunohistochemistry or immunofluorescence.

Sample sections were blocked in 5% normal donkey serum, which was diluted in 0.3% Triton X-100 PBS buffer, for 2 hours and then incubated in polyclonal primary antibodies to tyrosine hydroxylase (TH) (Pel-Freez, 1 : 1000 dilution) at 4°C for 48 hours. After several washes, tissue sections were further probed with biotinylated secondary antibodies (Chemicon, 1 : 200 dilution) for 2 hours and then ABC reagents (Beyotime, 1 : 500 dilution) for 30 mins, applying DAB to each section for staining and immersing into ddH_2_O when attaining acceptable staining intensity and, finally, dehydrating sections and mounting them with coverslips. For double immunofluorescence staining, cryosections were blocked in PBS containing 5% normal donkey serum for 2 h at room temperature. Sections were incubated overnight at 4°C with the primary antibodies against GFAP, NF200 (mouse anti-neurofilament 200 kDa monoclonal antibody, 1 : 50, Chemicon), and CGRP (rabbit anti-calcitonin gene-related peptide antibody, 1 : 2000, Sigma). After being washed with PBS 3 times, the sections were incubated for 1 h at room temperature with Alexa Fluor 488-labeled donkey anti-rabbit IgG (1 : 400, Life Technologies) and Alexa Fluor 594-labeled donkey anti-mouse IgG (1 : 400, Life Technologies). Sections were washed 3 times in PBS, followed by mounting tissue with the Dako Fluorescence Mounting Medium.

As the sprouting of postganglionic sympathetic fibers in DRG is believed to be induced by neurotrophin, we also detected the distributions of two types of neurotrophin membrane receptors: low-affinity p75 receptor (p75NTR) and high-affinity tropomyosin-related kinase A receptor (TrkA). Sections were incubated with the primary antibodies against p75 (rabbit anti-p75 NGF receptor antibody, 1 : 50, Abcam) or TrkA (rabbit anti-TrkA antibody, 1 : 1000, Abcam). After being washed with PBS 3 times, the sections were incubated for 1 h at room temperature with Alexa Fluor 594-labeled donkey anti-rabbit IgG (1 : 400, Life technologies).

### 2.7. Quantification of TH-IR Fibers

Six L5 DRG sections per sample at each time point were stained for TH-IR (immunoreactive) fiber counting. Details were described previously [36, 37]. Images of DRG sections were divided into cell body-concentrated region and TH-IR axon-focused region. For adult rats, 4 micrographs from the cell body region and 2 from the axon region were randomly taken per sample under 200x magnification. The length of TH-IR fibers was measured by using the NIH Image analysis program. We only counted visible fibers; fibers on blood vessels or oblique cutting were excluded. For infant rats, micrographs were selected under 400x magnification in sections collected before postoperative day 28; sections from days 28 to 56 were conducted the same with the adult rats.

### 2.8. Statistical Analysis

Statistical analyses were performed using SPSS 22.0 software (IBM, Armonk, NY, USA). *T*-test and ANOVA followed by LSD test were used to compare the behavior parameters between the two groups and among different time points after surgery. *T*-test was also carried out to analyze the differences in the density of sympathetic nerve fibers between the surgery group and the sham group. *P* < 0.05 was considered as statistically significant.

## 3. Results

L5 SNL surgery induces delayed-onset mechanical allodynia in infant rats.

**Figure 1:**
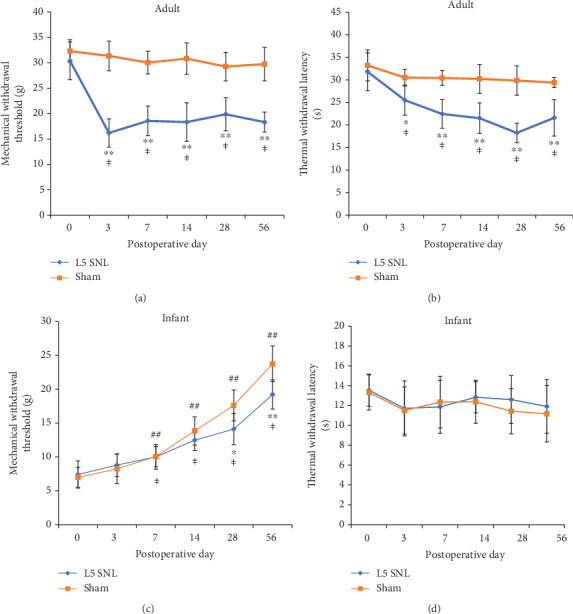
L5 SNL in adult rats produces the early mechanical and thermal (50°C) pain hypersensitivity (a, b). Infant rats show delayed-onset mechanical allodynia from postoperative day 28 (c). No heat hyperalgesia was observed in pups (d). SNL surgery: *n* = 10, sham control: *n* = 6; ^†^*P* < 0.05 vs. preoperative test in L5 SNL group and ^‡^*P* < 0.01; ^##^*P* < 0.01 vs. preoperative test in sham group, one-way ANOVA; ^∗^*P* < 0.05 vs. sham control and ^∗∗^*P* < 0.01, Student's *t*-test.

L5 SNL surgery in adult rats causes a dramatic and statistically significant reduction of mechanical withdrawal threshold and heat latency on the ipsilateral hind paw from postoperative day 3 and persisted to day 56 (Figures 1(a) and 1(b)). In contrast, the mechanical threshold of the pups shows no difference compared to that of the sham controls before postoperative day 28, though the values gradually increase with the postoperative time. From day 28, the mechanical withdrawal threshold of SNL groups in infant rats begins to be significantly lower than that of sham groups, which indicates that delayed-onset mechanical allodynia is developed (Figure 1(c)). No significant difference in thermal withdrawal latency is observed in infant rats at each postoperative time point between the two groups (Figure 1(d)).

Sympathetic sprouting fibers follow a similar time course with the development of mechanical allodynia after L5 SNL in pups.

**Figure 2:**
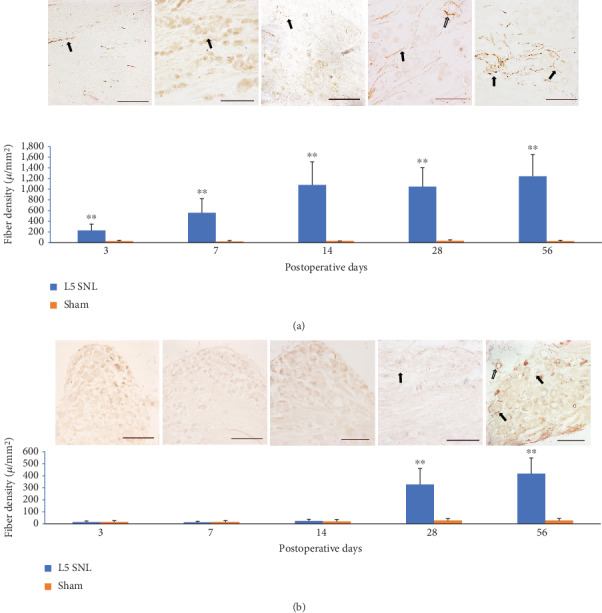
Adult nerve injury causes abnormal sprouting of TH-IR fibers in L5 DRGs at all postoperative time points. Some fibers form the ring-like structures around the neurons (a). Infant rats do not show the significant sprouting of TH-IR fibers until postoperative day 28 (b). SNL surgery: *n* = 6, sham control: *n* = 3; ^∗∗^*P* < 0.01 vs. sham control, Student's *t*-test; scale bars: 100 *μ*m; solid arrows: sympathetic sprouting; hollow arrow: perivascular sympathetic plexus.

In adult rats, the density of TH-IR sympathetic fibers in L5 DRG is progressively increased since postoperative day 3 and kept a high level from days 14 to 56. Sprouting fibers distribute extensively in both the cellular and axonal regions of the DRG. Some fibers form the ring-like structures around neurons. At any time point after adult L5 SNL, the density of TH-IR fibers in L5 DRG of SNL animals is significantly higher than that in the sham surgery group, of which the sprouting fibers are only occasionally detected, and no ring-like structure is observed (Figure 2(a)).

In pups, the TH-IR fibers are very scarce within 14 days after surgery. From days 28, the sprouting fibers begin to increase substantially in the entire region of the DRG, some of which also form the ring-like structures around the neurons, and persist to day 56 (Figure 2(b)).

**Figure 3:**
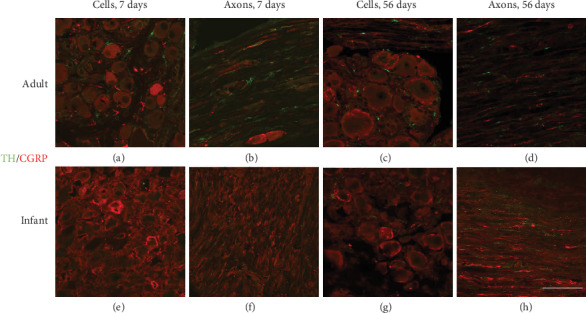
In L5 DRG of adult rats, TH-IR (green) fibers widely sprout around the CGRP (red)-positive neurons or axons after surgery (a–d). Infant rats show the delayed sprouting TH-IR fibers adjacent to neurons or axons expressing CGRP. Scale bars: 100 *μ*m.

**Figure 4:**
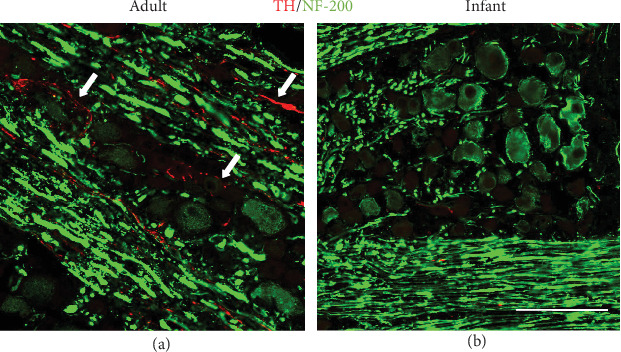
TH-IR (red) fiber sprout around the neurons or axons expressing NF-200 (green) in adult rats (a) but not in pups (b) at postoperative day 7. Arrows: the sprouting TH-IR fibers. Scale bars: 50 *μ*m.

The double immunofluorescence staining confirms that the TH-IR rings are usually formed around the primary afferent neurons expressing CGRP (Figure 3) or NF-200 (Figure 4) in both adult and pup rats.

### 3.1. p75NTR Expression

**Figure 5:**
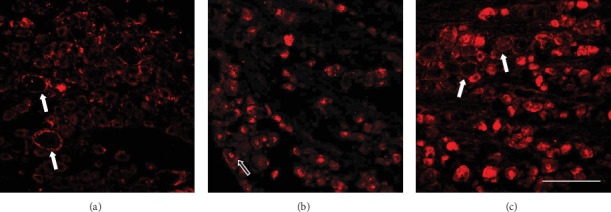
The glia cell rings expressing p75NTR were presented around the neurons of DRG in adult rats (a) but not in pups (b) at postoperative day 7. Glial p75NTR was observed at postoperative day 28 in pups (c). Solid arrows: SCG rings expressing p75NTR; hollow arrow: neurons expressing p75NTR at the nucleus. Scale bars: 100 *μ*m.

Recent studies have revealed that neurotrophin receptors, which are mainly expressed in the satellite glial cells (SGCs) after nerve injury, play an essential role in the development of sympathetic sprouting [38–40]. Thus, we evaluated the expression of the low-affinity receptor p75NTR and the high-affinity receptor TrkA in ipsilateral L5 DRG neurons. Typically, the p75NTR only distributed in neuronal cytoplasm in DRG of both adult and pup rats. Adult nerve injuries produce the formation of rings of astrocytes, which express both receptors highly, from the early postoperative stage (Figure 5(a)). In contrast, the infant nerve injuries do not induce the increasing of the p75NTR-IR glial around the neurons within postoperative day 28. There is also a high expression of p75NTR in the nuclear envelope (Figures 5(b) and 5(c)). From day 28, the pup rats also showed a delayed-onset presence of p75NTR-IR glial rings, which is parallel to the occurrence of mechanical hyperalgesia. The infant nerve injury did not induce the glial ring to highly expressing TrkA at any postoperative time point (Supplementary Figure 1).

## 4. Discussion

In this study, we compared the long-term consequences of L5 SNL on pain behaviors between infant and adult rats. In contrast to the rapid development of the pain behaviors in adults, the pups did not show mechanical allodynia until 28 days after nerve injury. The mechanical withdrawal threshold steadily increased with postnatal development, as previously described [6, 41]. Therefore, the actual postoperative changes of the mechanical threshold of the pups are mixed up with the decrease in the threshold caused by the surgery and the normal developmental increase. To overcome this issue, we compared the measurement between SNL and sham groups at each time point to identify the real effect of the surgery on the pain behaviors. From postoperative day 28, however, the rising slowed down on the injured side, with the threshold value significantly lower than that of the sham control, suggesting the mechanical pain sensitivity was produced in pups. No difference was observed in heat latency value between the two groups at any postoperative time points in pups, suggesting nerve lesion in early life has a specific effect on adolescent mechanical nociception. In the spared nerve injury (SNI) model, the mechanical but not heat pain behaviors were developed in infant rats at 21 days after surgery [10, 12]. Unlike the above two models, however, a tail-innervating nerve lesion in the pups induced a less persistent mechanical and cold allodynia from 6 to 8 weeks after surgery [11]. The various characteristics of pain behavior may be attributed to the type of nerve injury. In this study, the delayed-onset mechanical allodynia of pups with L5 SNL may provide an opportunity to further investigate the mechanisms involving in development of neuropathic pain.

The sympathetic sprouting fiber in DRG has been described as a significant event involving in the development of neuropathic pain following L5 SNL following adult nerve lesion [16, 19, 20]. The sprouting fibers preferentially formed nests with synapse-like structures around the spontaneously active neurons or tangled with efferent fibers [18, 42]. The number of sprouting fibers had been proved statistically correlated with neuropathic behaviors [16, 43]. Synapse-like structures made by sympathetic fibers and dendrites were proved to contribute to the maintenance of chronic pain state [18]. The reduced sprouting produced by early fiber blockage decreased the spontaneous activity of DRG neurons and pain behaviors [43–45]. All the previous results were achieved from adult neuropathic pain models. In this study, the pups showed a higher density of sympathetic fibers than the sham controls from 28 days after surgery. To our knowledge, it is the first time to demonstrate delayed-onset sympathetic sprouting in DRG of animal models with early nerve lesions. Although not as high as that in adult rats, the density of sprouting fibers significantly increased as compared with the sham control during the adolescent period. The time course is parallel to the occurrence of mechanical allodynia behavior in pups. These results suggest that the delayed-onset sprouting of sympathetic fibers may be involved in the presence of neuropathic pain in adolescence in case of nerve injury in infancy and childhood. Recently, accumulating studies have targeted on reducing the sprouting of sympathetic fibers to relieve the neuropathic pain symptom. Dexmedetomidine, a selectively strong agonist of the *α*2-adrenergic receptor, was proved to alleviate neuropathic pain by decreasing the sympathetic sprouting. Future studies targeting mechanism blocking the sympathetic sprouting may provide a novel therapeutic strategy in the prevention or treatment of delayed neuropathic pain in adolescence following nerve injury in an early stage.

To identify the possible mechanisms involving in the absence of early sympathetic sprouting for pups, we conducted a series of comparative immunohistochemical and immunofluorescence analysis between adult and pup rats, including the SGCs (glial fibrillary acidic protein) (Supplementary Figure 2), macrophages (OX-42, major histocompatibility complex-II), nerve growth factor (Supplementary Figures 3 and 4), and neurotrophin receptors (p75NTR, TrkA). Only the distribution of glial p75NTR showed a significant difference between the two-age groups at the early postoperative period. Neurotrophins level plays a crucial role in sympathetic sprouting as well as the generation of neuropathic pain [37, 46]. After adult nerve injury, synthesis of neurotrophins in SGCs surrounding lesioned neurons elevated, which activate the p75NTR or Trk receptors to induce neuropathic pain [38, 47–50]. As a low-affinity neurotrophin receptor, p75NTR was confirmed to be essential for sympathetic sprouting caused by elevated neurotrophins levels [40]. Knockout of the gene could significantly reduce sympathetic sprouting after nerve injury, which confirmed a causal relationship among neurotrophins, p75NTR, and sympathetic sprouting in adult nerve lesions [51, 52]. In this study, we observed a delayed high expression of glia p75NTR around the neurons 28 days after infant nerve lesion, and the change is precisely parallel to the onset of sympathetic sprouting. The results suggested that the mechanism by which the sympathetic sprouting delayed may attribute to the late increase of p75NTR expression around neurons after nerve injury in the early postnatal stage. Astrocytes in the cat visual cortex were observed to develop a mature stellate appearance between the third and seventh postnatal weeks [53]. The receptors regulating neuron-SGC communication also showed evident postnatal changes in DRGs [54]. We speculate that the early absence of p75NTR expression may be relative to the immaturity of SGCs and the different neuron-SGC communications from the adult ones. With the maturation of SGCs, the expression of p75NTR increases gradually, and sympathetic axons are attracted to sprout towards the neurons. The absent parallel increase expression of TrkA receptor suggests that TrkA may not directly contribute to the development of mechanical allodynia in the infant nerve lesion. These results might provide an insight into the discovery of novel ways to improve the preventive or therapeutic effects on delayed-onset neuropathic pain after an early period of nerve injury. For example, p75NTR inhibitors might be developed to selectively block the neurotrophin signaling to reduce the risk of neuropathic pain in adolescence.

In conclusion, we showed a delayed onset of mechanical allodynia in the infant after L5 SNL surgery accompanied with sympathetic sprouting in DRG. The delayed expression of glial p75NTR may play an important role in the presence of sympathetic sprouting as well as neuropathic pain. Further studies targeting the mechanisms underlying the relationship between p75NTR and the sympathetic sprouting in the infant may provide a novel therapeutic strategy in the treatment of neuropathic pain.

## Data Availability

The data used to support the findings of this study are included within the article and the supplementary information files.

## Supplementary Materials

Supplementary Materials Supplementary Figure 1: the glia cell rings expressing TrkA are presented around the neurons of DRG in adult rats (a) but not in pups at postoperative day 7 (b). Solid arrows, the glia cell rings expressing TrkA. Scale bars, 100 *μ*m. Supplementary Figure 2: immunofluorescence staining for GFAP of dorsal root ganglion 3 days after L5 SNL in adult (a) and pup rats (b). There were activated GFAP(+) satellite glial cells (arrows) around the neurons in both groups, mainly the large ones. Supplementary Figure 3: double staining for NGF (red) and MHCII (green) of dorsal root ganglion 7 days after L5 SNL in adult (a) and pup rats (b). Both groups showed the infiltration of MHCII(+) macrophages (arrows) surrounding the neurons. Supplementary Figure 4: immunohistochemical staining for OX-42 of dorsal root ganglion 7ays after L5 SNL in adult (a) and pup rats (b). Both groups showed the infiltration of OX-42(+) macrophages (arrows) surrounding the neurons.
