# Supplementary material for: The Delayed-Onset Mechanical Pain Behavior Induced by Infant Peripheral Nerve Injury Is Accompanied by Sympathetic Sprouting in the Dorsal Root Ganglion
Source: Biomed Res Int. 2020 Jun 16;2020:9165475. doi: 10.1155/2020/9165475 (PMC7315272; doi:10.1155/2020/9165475)

**Supplementary Figure Legends**

Supplementary Figure 1. The glia cell rings expressing TrkA are presented around the neurons of DRG in adult rats (a) but not in pups at postoperative day 7 (b). Solid arrows, the glia cell rings expressing TrkA. Scale bars, 100 µm.

Supplementary Figure 2. [Immunofluorescence](javascript:;) staining for GFAP of dorsal root ganglion 3 days after L5 SNL in adult (a) and pup rats (b)­­. There were activated GFAP (+) satellite glial cells (arrows) around the neurons in both groups, mainly the large ones.

Supplementary Figure 3. Double staining for NGF (red) and MHCII (green) of dorsal root ganglion 7 days after L5 SNL in adult (a) and pup rats (b)­­. Both groups showed the infiltration of MHCII(+) macrophages (arrows) surroundingthe neurons.

Supplementary Figure 4. Immunohistochemical staining for OX-42 of dorsal root ganglion 7ays after L5 SNL in adult (a) and pup rats (b)­­. Both groups showed the infiltration of OX-42(+) macrophages (arrows) surroundingthe neurons.


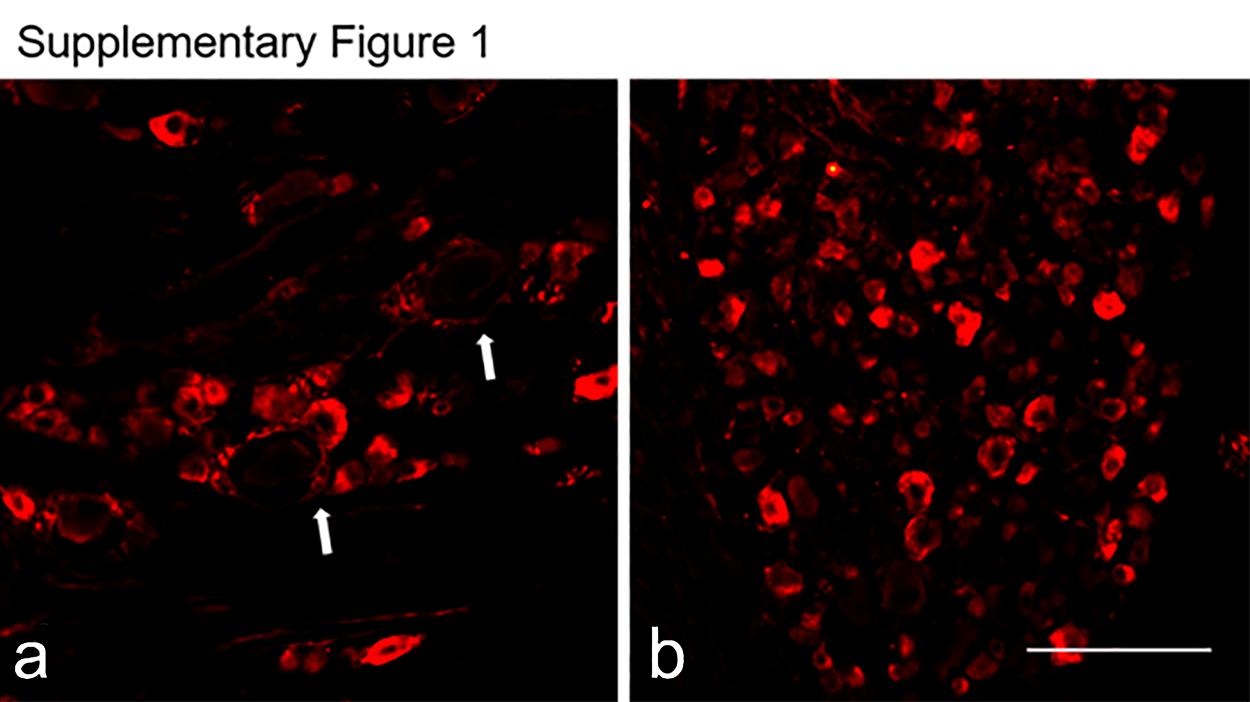


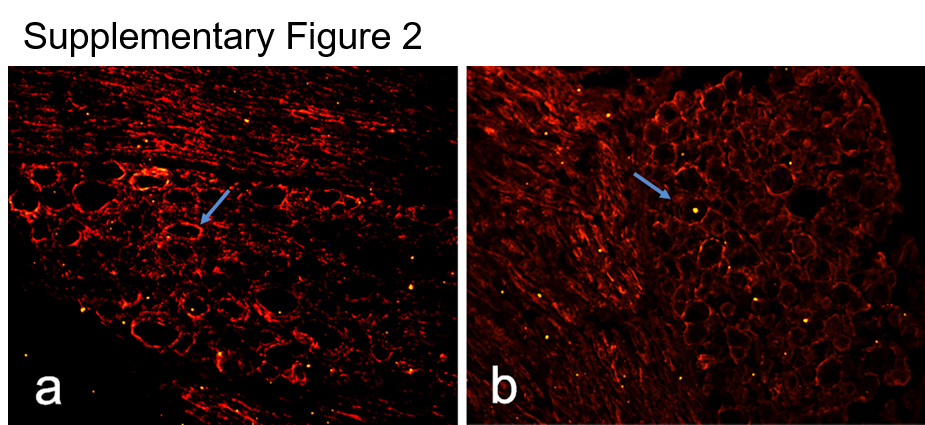

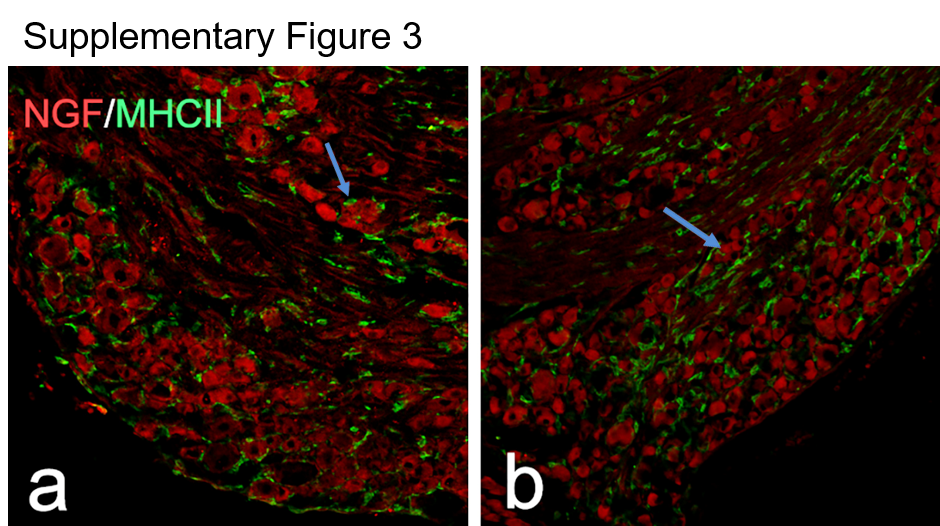

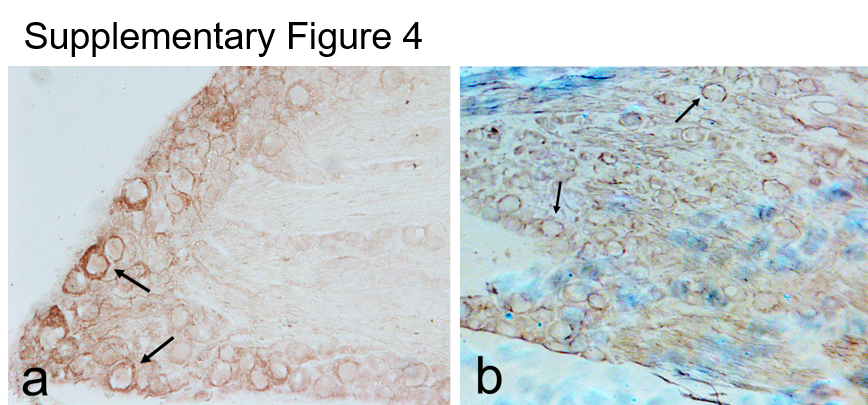

Supplement: Supplementary Materials — Supplementary Figure 1: the glia cell rings expressing TrkA are presented around the neurons of DRG in adult rats (a) but not in pups at postoperative day 7 (b). Solid arrows, the glia cell rings expressing TrkA. Scale bars, 100 μm. Supplementary Figure 2: immunofluorescence staining for GFAP of dorsal root ganglion 3 days after L5 SNL in adult (a) and pup rats (b). There were activated GFAP(+) satellite glial cells (arrows) around the neurons in both groups, mainly the large ones. Supplementary Figure 3: double staining for NGF (red) and MHCII (green) of dorsal root ganglion 7 days after L5 SNL in adult (a) and pup rats (b). Both groups showed the infiltration of MHCII(+) macrophages (arrows) surrounding the neurons. Supplementary Figure 4: immunohistochemical staining for OX-42 of dorsal root ganglion 7ays after L5 SNL in adult (a) and pup rats (b). Both groups showed the infiltration of OX-42(+) macrophages (arrows) surrounding the neurons. [file 9165475.f1.docx]
